# Two phenotypes of chronic recurrent multifocal osteomyelitis with different patterns of bone involvement

**DOI:** 10.1186/s12969-022-00772-w

**Published:** 2022-12-01

**Authors:** Dita Cebecauerová, Hana Malcová, Veronika Koukolská, Zuzana Kvíčalová, Ondřej Souček, Lukáš Wagenknecht, Jiří Bronský, Zdeněk Šumník, Martin Kynčl, Marek Cebecauer, Rudolf Horváth

**Affiliations:** 1 Department of Paediatric and Adult Rheumatology, Motol University Hospital, Prague, Czech Republic; 2 Department of Radiology, Second Faculty of Medicine, Charles University and Motol University Hospital, Prague, Czech Republic; 3 Department of Biophysical Chemistry, J. Heyrovsky Institute of Physical Chemistry of the Czech Academy of Sciences, Prague, Czech Republic; 4 Department of Pediatrics, Second Faculty of Medicine, Charles University and Motol University Hospital, Prague, Czech Republic; 5 Department of Ortopaedics, Second Faculty of Medicine, Motol University Hospital, Prague, Czech Republic

**Keywords:** Autoinflammatory disease, Chronic non-bacterial osteomyelitis, Chronic recurrent multifocal osteomyelitis, Inflammatory bowel disease, Sacroiliitis, Whole-body magnetic resonance imaging

## Abstract

**Introduction:**

Chronic Recurrent Multifocal Osteomyelitis (CRMO) is an autoinflammatory bone disorder with predominantly paediatric onset. Children present with multifocal osteolytic lesions accompanied by bone pain and soft tissue swelling. Patients often exhibit extraosseous co-morbidities such as psoriasis, inflammatory bowel disease, and arthritis.

**Objectives:**

Comparison of children with two different phenotypes of CRMO defined by presence or absence of extraosseous co-morbidities.

**Methods:**

Children diagnosed with CRMO at the Motol University Hospital between 2010 and 2020 were retrospectively reviewed, and according to the absence or presence of extraosseous manifestations divided into two cohorts – bone limited CRMO and complex CRMO. The two groups were compared in terms of demographic data, age at disease onset, number and site of bone lesions, laboratory biomarker values, and need of escalation to a second-line therapy.

**Results:**

Thirty-seven children (30 female, 7 male) with confirmed CRMO were included in the analysis. The mean age at disease onset was 10 years. All but 3 patients presented with multifocal disease. Twenty-three children (62%) had at least one extraosseous manifestation (13 sacroiliitis, 8 inflammatory bowel disease, 6 skin disease [acne, pustulosis, or psoriasis], 7 arthritis). Complex CRMO was associated with a significantly higher ESR rate (*p* = 0.0064) and CRP level (*p* = 0.018). The groups did not differ in number of foci or in age at disease onset. Bone lesion distribution differed between the two groups with significantly more frequent involvement of clavicle (*p* = 0.011) and pelvis (*p* = 0.038) in patients with complex CRMO. Children with complex CRMO more often needed escalation of therapy to DMARDs and biologic agents.

**Conclusion:**

Our data suggest that CRMO affecting solely the skeleton has milder course compared to complex CRMO with extraskeletal features. Further studies are needed to explore the clinical as well as the patient reported outcomes and promote individually tailored therapeutic strategies in both CRMO phenotypes.

## Introduction

Chronic Recurrent Multifocal Osteomyelitis (CRMO) is a rare autoinflammatory disorder characterized by sterile bone lesions [1, 2]. It is often referred to as a subgroup within Chronic Non-Bacterial Osteomyelitis (CNO). For patients with concomitant acne or pustulosis the acronym SAPHO (Synovitis, acne, pustulosis, hyperostosis, osteitis) is used. CRMO incidence is estimated at 1:250000 [3]; precise values remain unknown. However, the number of diagnosed cases has increased lately with improved public and care-provider awareness and better accessibility of whole-body magnetic resonance imaging (WB-MRI) [4, 5]. It affects predominantly young girls with peak presentation at age 10 years [6–8], typically with bone pain accompanied by local warmth and soft tissue swelling. The disease course is recurrent or persistent with a variable number of osteolytic lesions developing over time. The unifocal and persistent form is more common in adult patients [9]. Adults and adolescents often display involvement of clavicle and sternum and have various cutaneous manifestations [9, 10].

The molecular pathogenesis is not fully understood, but there is a consensus that CRMO is an autoinflammatory disease, characterized by unprovoked spontaneous flares of tissue-specific bone inflammation. The fact that different autoimmune conditions frequently affect pedigrees of CRMO patients emphasizes the role of genetic predispositions. However, specific genetic variants are characterised only in a small proportion of patients [11–14]. Typical examples of monogenic forms of osteomyelitis represent Majeed syndrome or DIRA (Deficiency of Interleukin-1 Receptor Antagonist), with an altered IL-1 signalling pathway. Mutations in the coding and regulatory parts of *FLBIM1* are found in a minority of children with non-syndromic CRMO together with an altered IL-10 signalling pathway [11, 12]. These variants are, nevertheless, considered as contributing to rather than as underlying CRMO. At this writing (rev. Sept 2022), no other entries for CRMO exist in the Online Mendelian Inheritance in Man database (https://omim.org/). Clinically, CRMO shares significant overlap with TNFα-related autoimmune conditions such as spondyloarthritis, psoriasis, and inflammatory bowel disease (IBD). Indeed, CRMO and/or SAPHO syndrome may, over time, evolve into spondyloarthropathy in some patients [15]. The available data are limited but suggest that aetiopathogenesis of CRMO is complex and that probably heterogenous genetic background underlies a seemingly uniform phenotype.

Recent reports indicate that about half of CRMO patients have associated extraosseous involvement, mostly psoriasis and palmo-plantar pustulosis, IBD, and arthritis. The frequency of particular co-morbidities varies significantly among reported cohorts [6, 8, 9, 16, 17]. Whilst cutaneous involvement is reported in 10–30% of patients, IBD is diagnosed in a much smaller group (5–13%). Arthritis varies significantly between individual cohorts (6–56%). Evidently, more studies are required better to understand these complex relations between CRMO and associated conditions.

So far, no specific guidelines exist for the treatment of children with CRMO. Therapy response assessment is complicated by the lack of a definition of disease remission. Based on current treatment practices, Zhao et al. published consensus treatment plans for CRMO refractory to non-steroidal anti-inflammatory drugs (NSAIDs) [10]. Whereas NSAIDs represent first-line treatment, bisphosphonates are used in case of spinal involvement or NSAIDs failure. Disease-modifying anti-rheumatic drugs (DMARDs) and short-course corticosteroids can be used to escalate therapy; however, disease remission is achieved in fewer patients for whom these approaches are required [6, 9, 18, 19]. Biologic DMARDs, primarily tumour necrosis factor α inhibitors (TNFi), are the preferred choice in refractory disease or associated conditions, including sacroiliitis or IBD [20, 21].

This study postulated that the two distinct phenotypic manifestations of CRMO – a solely osseous form, with only bone involvement, and a complex form, characterised by the presence of extraosseous associated conditions – might differ in clinical and laboratory aspects of disease, and sought to compare cohorts of children with the two forms of CRMO.

## Methods

We retrospectively reviewed and analysed a cohort of paediatric CRMO patients diagnosed and treated at Motol University Hospital, Prague, between 2010 and 2020. Only children still followed-up in Motol University Hospital, were included in the study. The diagnosis was based on the typical clinical and radiological findings, negative microbiology results and in 62% of children was completed by histologic confirmation of chronic non-bacterial osteomyelitis. Routine histopathology assessment and description was provided by several independent histopathologists. The retrospective assessment of histopathology description did not reveal any differences between the two groups of patients. No retrospective assessment of specimens has been performed. Bristol criteria [16, 22] were applied retrospectively to all patients included in the study. The study was approved by the local Ethics Committee (EK 1412/20). Written informed consent was obtained from patients or legal guardians before study enrolment. From medical records we collected clinical data, including family history, gender, ethnicity, age of disease onset, first symptoms at manifestation, number and distribution of bone lesions, biopsy results, microbiologic results, spinal involvement, inflammatory and immunological marker values, faecal calprotectin levels, extraosseous disease, and therapy. WB-MRI and X-ray imaging were re-assessed by an experienced radiologist. Sacroiliac-joint involvement was diagnosed only when typical oedema of subchondral bone was present. In case WB-MRI was unavailable (in 4 out of 37 children), the results from both, MRI and bone scintigraphy were assessed.

### Statistical analysis

Fisher’s exact test was used to compare the two groups of patients with lesions present and absent at a given site. All datasets (age at onset, number of foci, erythrocyte sedimentation rate [ESR], and serum C-reactive protein [CRP]) were first tested for normality using quantile-quantile plots and the Anderson-Darling test. Number of lesions and age at onset showed normal distributions with equal variance (two-sample F-test). Two-sample unpaired Student’s t-testing was used to compare the means between the bone limited and complex CRMO groups. For ESR and CRP parameters not following a normal distribution, means were compared with the two-sample Kolmogorov-Smirnoff test. Statistical analyses were performed using appropriate functions of MATLAB software (Mathworks, Natick, MA).

## Results

**Fig. 1:**
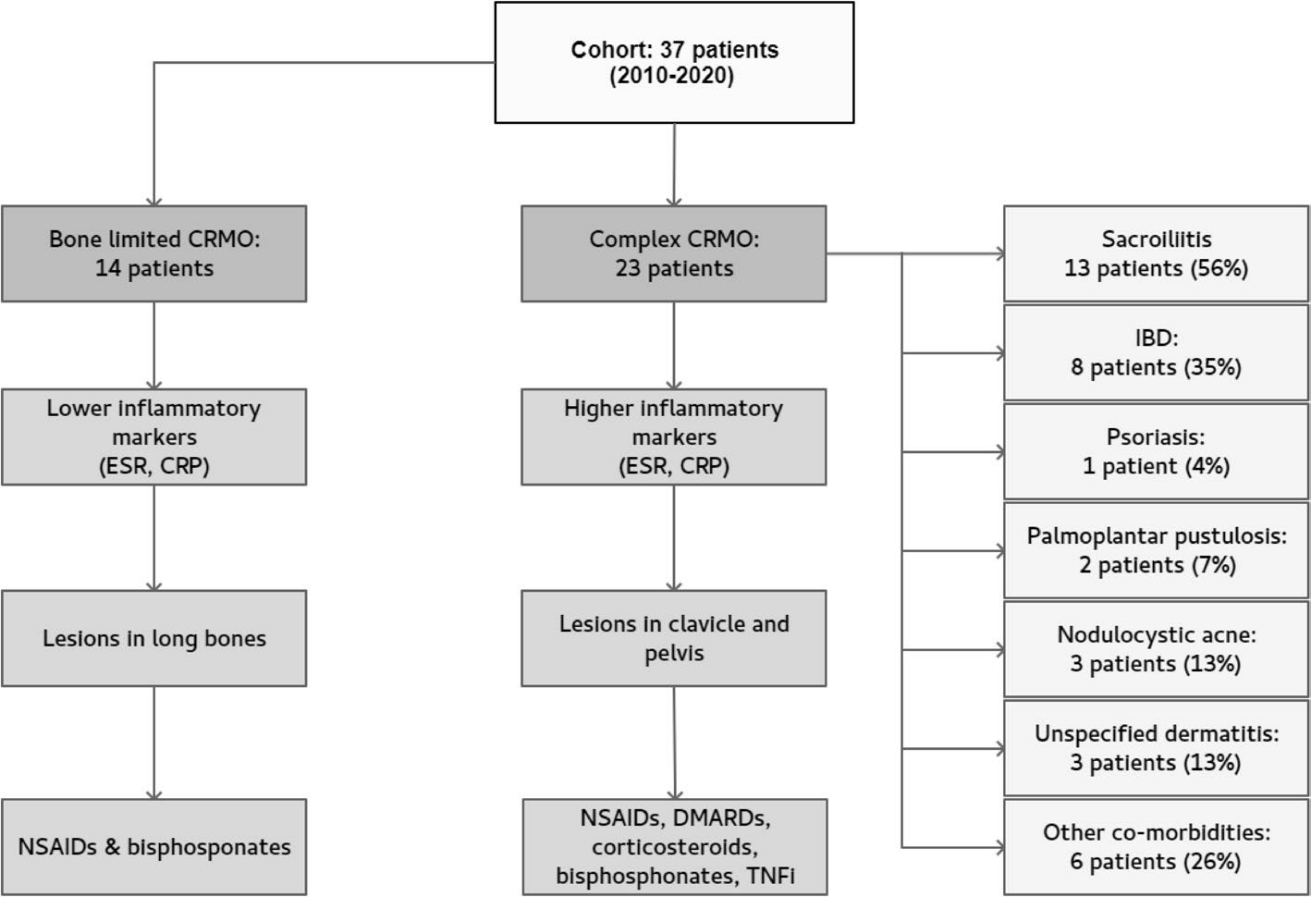
A graphical scheme summarising critical findings in our cohort. CRP – C-reactive protein, DMARD - disease-modifying antirheumatic drugs, ESR - erythrocyte sedimentation rate, IBD – inflammatory bowel disease, NSAID - non-steroidal anti-inflammatory drugs, TNFi – tumour necrosis factor α inhibitors

Thirty-seven children, 30 girls (81%) and 7 boys (19%), were enrolled in the study (Fig. 1). Their clinical characteristics are summarized in Table 1. The initial symptom in most cases was limb pain (51%), followed by back pain (38%). The prevalently affected site was pelvis (59%), followed by femur (49%), tibia (43%), spine (38%), and clavicle (35%). Clavicular swelling as a first sign was present in 2 patients (5%); however, WB-MRI revealed asymptomatic clavicular lesions in 11 more children, with bilateral involvement in 4. Diaphyseal regions were affected in 9 children (24%). In six the lesion laid within the large metaphyseal-diaphyseal region. Solely diaphyseal lesions were confirmed in 4 children (11%). The least involved regions were skull, mandible, and scapula, with involvement in 2 children each. Just 3 patients (8%) were proven to have a single osteolytic site on WB-MRI. These solitary lesions were in 10th thoracic vertebra, clavicle, and humerus.

**Table 1:** Demographic and clinical characteristics of the cohort.

Total number of patients	37
**Female**	30 (81%)
**Male**	7 (19%)
**Ethnicity**	
**Romani**	4 (11%)
**Other Czech**	33 (89%)
**Age at onset, years; median (range)**	10 (4.6–17.3)
**Number of lesions; median (range)**	7 (1–19)
**Skeletal involvement**	
**Unifocal**	3 (8%)
**Multifocal**	34 (92%)
**Initial symptom**	
**- Back pain**	14 (38%)
**- Limb pain**	19 (51%)
**- Other**	4 (11%)
**Bone biopsy**	23 (62%)
**Spinal lesions**	14 (38%)
**- Spinal surgery**	7 (19%)
**Sacroiliac joint involvement**	13 (35%)
**Inflammatory bowel disease**	8 (22%)
**Psoriasis / palmoplantar pustulosis**	3 (8%)
**Nodulocystic acne**	3 (8%)
**Unspecific dermatitis involvement**	3 (8%)
**Autoimmune comorbidities in family history**	25 (68%)
**- First-degree (I) relatives**	15
**- Second-degree (II) relatives**	13
**Anti-nuclear antibodies present**	2 (5%)
**HLA-B27 present**	6 (16%)

Inflammation-marker values were elevated at presentation in most of the patients. ESR and/or CRP were increased in 24 children (65%) at the time of diagnosis, but they did not intercorrelate in all patients, i.e., some children had elevated ESR but not CRP and vice versa. Only 5 children had inflammatory-marker values within normal range. ANA were identified in 2 patients (5%). HLA-B27 positivity was detected in 6 children (16%).

**Fig. 2:**
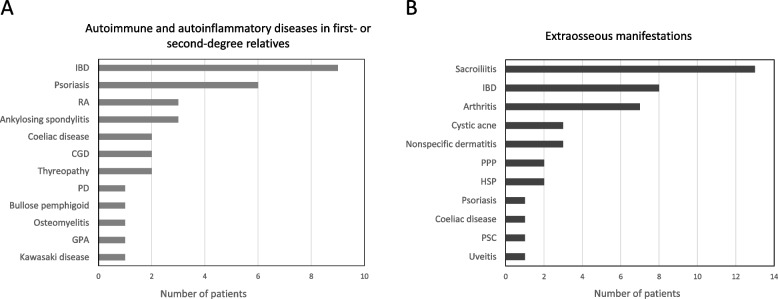
Autoimmunity profiles. **A** Summary of total autoimmune disease manifestations in the first- and second-degree relatives and of **B** extraosseous comorbidities in CRMO patients. CGD – chronic granulomatous disease, GPA – granulomatosis with polyangiitis, HSP – Henoch-Schönlein purpura, IBD – inflammatory bowel disease, PD – phagocytic dysfunction, PPP – palmoplantar pustulosis, PSC – primary biliary cirrhosis RA – rheumatoid arthritis

**Fig. 3:**
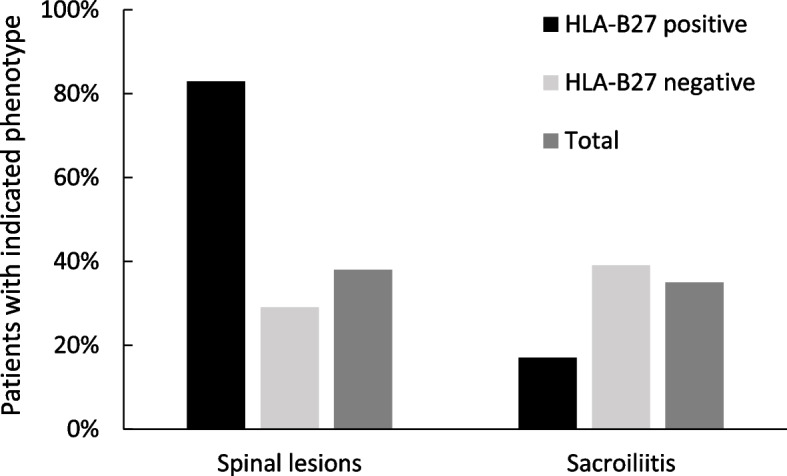
Vertebral and sacroiliac involvement. HLA-B27 positive children with CRMO displayed sacroiliac involvement less frequently than spinal involvement. Vertebral lesions were twice as frequent as in the cohort. Sacroiliitis in HLA-B27 positive CRMO was less frequent than sacroiliitis in HLA-B27 negative CRMO children and in non-selected CRMO children

Twenty-five children (68%) had family histories of one or more autoimmune or autoinflammatory diseases in first- and second-degree relatives (Fig. 2A), most often IBD (6) and psoriasis (3). Twenty-three CRMO patients (62%) had associated extraosseous conditions (Fig. 2B) at the time of diagnosis or developed them during follow-up (1–10 years). These were considered as ‘complex CRMO’ in this study. Thirteen children (35%) had sacroiliitis (Table 1), 7 of them symptomatic. In 6 patients, sacroiliitis remained clinically silent. Bilateral sacroiliitis (9 children) was more frequent than unilateral (4 children) and was not associated with HLA-B27 positivity. Only one HLA-B27 positive child had MRI-proved sacroiliitis (Fig. 3 and Table 2). In this patient, sacroiliitis remained asymptomatic despite ongoing inflammation detectable by MRI. Three children had positive family history of IBD in first- or second- degree relatives, but not HLA-B27 positive spondyloarthritis (SpA). Four children presented with elevated levels of faecal calprotectin (FCP); of those, only one developed IBD during follow-up (3–12 years).

**Table 2:** Characteristics of patients with HLA-B27 positive CRMO

No.	Gender	Sacroiliitis	Spine	CRMO subtype	FCP (μg/g)	ASCA/ANCA	Family history	Co-morbidity
**1.**	F	no	yes	bone limited	< 50	ASCA positive	negative	none
**2.**	F	no	yes	complex	350	ASCA positive	I.IBD	IBD (CD), HSP, uveitis
**3.**	F	yes	no	complex	187	ASCA bordeline	negative	arthritis, sacroiliitis
**4.**	M	no	yes	bone limited	< 50	negative	negative	none
**5.**	F	no	yes	bone limited	125	negative	II.IBD	none
**6.**	F	no	yes	complex	230	negative	I. Coeliac disease, II.IBD	coeliac disease

*Abbreviations*: *ANCA* anti-neutrophil cytoplasmic antibody, *ASCA* anti-*Saccharomyces cerevisiae* antibody, *CD* Crohn disease, *CRMO* chronic recurrent multifocal osteomyelitis, *FCP* foetal calprotectin, *I* first-degree relative, *II* second-degree relative, *HLA-B27* human leucocyte antigen B27, *HSP* Henoch-Schőnlein purpura, *IBD* inflammatory bowel disease

Eight children in our cohort (22%) developed enteropathic CRMO, osteomyelitis associated with IBD. Four had family histories of IBD. Table 3 summarizes characteristic features and the sequential course of their disease. CRMO preceded IBD in 3 patients and IBD preceded CRMO in 5. The average delay between presentation and manifestation of the associated disorder was 4 years. Two children who initially presented with CRMO and in whom complete remission of bone disease was achieved later developed IBD without relapse of CRMO. All children with enteropathic CRMO had elevated faecal calprotectin levels (Table 3). Mild elevation of FCP (up to 250 μg/g) was found in samples from 9 other CRMO patients, but they did not progress to IBD, and their FCP levels dropped during follow-up. The FCP levels were assessed in all but one patient (data not shown).

**Table 3:** Characteristics of patients with enteropathic chronic recurrent multifocal osteomyelitis

No.	Gender	No. foci	Autoantibodies	Histology	Disease course	Delay (years)	Therapy	Outcome	HLA-B27	FCP (μg/g)	Family history
1.	F	**7**	**ASCA positive**	**NS colitis**	**1. CRMO, arthritis** **2. colitis + CRMO relapse**	**5.0**	**SSZ -- > P-- > ADA + MTX -- > none**	**remission, now without therapy**	negative	**1000**	II. HLA-B27 AS
2.	M	**10**	**p-ANCA positive**	**CD**	**1. IBD** **2. PPP** **3. CRMO + IBD relapse**	**4.0**	**AZA, IFX, IFX + MTX -- > ADA**	**relapsing IBD and CRMO**	negative	**1736**	I. IBD
3.	F	**2**	**ASCA positive**	**CD**	**1. CRMO, arthritis** **2. IBD** **3. HSP** **4. acute uveitis**	**0.5**	**SSZ -- > ETA + MTX -- > ADA, AZA**	**remission on therapy**	positive	**350**	I. IBD
4.	F	**9**	**ASCA positive**	**CD**	**1. IBD** **2. CRMO + PPP**	**4.0**	**AZA -- > IFX-- > ADA-- > ADA + SSZ-- > ADA + MTX + SSZ**	**remission on therapy**	negative	**3329**	II. psoriasis
5.	F	**3**	**p-ANCA positive**	**UC**	**1. IBD** **2. IBD relaps + CRMO**	**2.5**	**P + AZA-- > 5-ASA-- > ADA + MTX**	**remission on therapy**	negative	**938**	negative
6.	F	**11**	**negative**	**UC, PSC**	**1. IBD, PSC** **2. CRMO**	**4.0**	**5-ASA, UDCA, PAM-- > ADA**	**active CRMO, IBD in remission**	negative	**1680**	II. IBD
7.	M	**7**	**p-ANCA positive**	**CD**	**1. CRMO, arthritis** **2. IBD (arthritis and** **CRMO in remission)**	**7.5**	**NSAID-- > SSZ, P, MTX -- > ADA**	**active IBD, CRMO in remission**	negative	**1230**	unknown
8.	M	**9**	**negative**	**CD**	**1. CRMO** **2. IBD (CRMO in remission)**	**4.0**	**NSAID-- > P-- > 5-ASA-- > non-compliance**	**active IBD, CRMO in remission**	negative	**545**	I. IBD, II. psoriasis

*Abbreviations*: *ADA* adalimumab, *ANCA* anti-neutrophil cytoplasmic antibody, *ASCA* anti-*Saccharomyces cerevisiae* antibody, *5-ASA* 5-aminosalicylic acid, *AZA* azathioprine, *CD* Crohn disease, *CRMO* chronic recurrent multifocal osteomyelitis, *ETA* etanercept, *FCP* foetal calprotectin, *I* first-degree relative, *II* second-degree relative, *HLA-B27* human leucocyte antigen B27, *HLA-B27 AS* HLA-B27 positive ankylosing spondylitis, *IBD* inflammatory bowel disease, *IFX* infliximab, *MTX* methotrexate, *NS colitis* non-specific colitis, *NSAID* non-steroidal anti-inflammatory drug, *P* prednisone, *PAM* pamidronate, *PPP* palmo-plantar pustulosis, *PSC* primary sclerosing cholangitis, *SSZ* sulphasalazine, *UC* ulcerative colitis, *UDCA* ursodeoxycholic acid

Arthritis was diagnosed in 7 children (19%); 6 of them had a family history of autoimmune disease. Arthritis primarily but not exclusively adjoined foci of osteomyelitis. Eight patients (22%) developed skin disease including acne (*n* = 3), pustular psoriasis (*n* = 1), palmoplantar pustulosis (*n* = 2), and atopic or unspecified dermatitis (n = 3). SAPHO syndrome was diagnosed in 3 individuals (8%) based on severe nodulocystic acne and clavicular involvement with typical imaging findings. All 3 patients had an inflammatory phenotype at the time of diagnosis. Palmoplantar pustulosis occurred as a paradoxical effect of TNFi therapy (1x infliximab, 1x adalimumab) in 2 children who initially suffered from IBD and later manifested CRMO. Their skin disease was managed by topical treatment without withdrawal of TNFi therapy. One patient presented with chronic remitting pustular dermatitis but was not diagnosed with psoriasis. Her skin disease continues into adulthood. Two different patients with enteropathic CRMO had atopic dermatitis and nail pitting, but not skin disease corresponding to psoriasis.

**Fig. 4:**
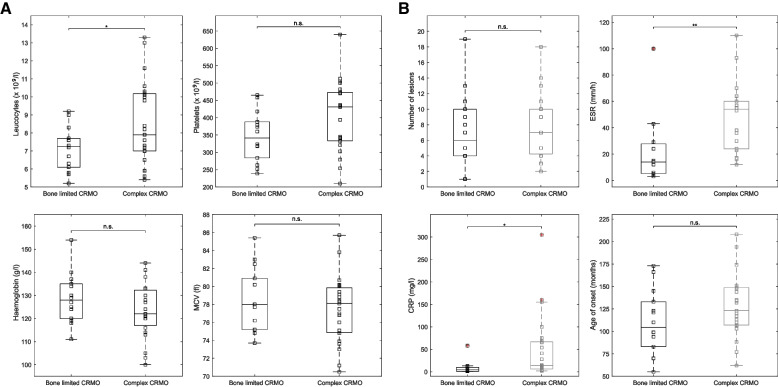
Comparison of laboratory characteristics between bone limited and complex CRMO patients. **A** Charts comparing blood markers, white blood count, platelets, haemoglobin, and mean cell volume (MCV) for the two groups of CRMO patients. Data are presented as boxplots with the 25th, 50th (median), and 75th percentiles marked. Whiskers extend to the most extreme data points. Squares represent individual patients. An asterisk (*) indicates statistical significance (*p* < 0.05). **B.** Comparison of inflammatory markers, number of lesions, and age at onset between bone limited and complex CRMO. Data are presented as boxplots with the 25th, 50th (median), and 75th percentiles marked. Whiskers extend to the most extreme data points. Squares represent individual patients. An asterisk (*) and two asterisks (**) indicate statistical significance (p < 0.05 and *p* < 0.01, respectively)

**Fig. 5:**
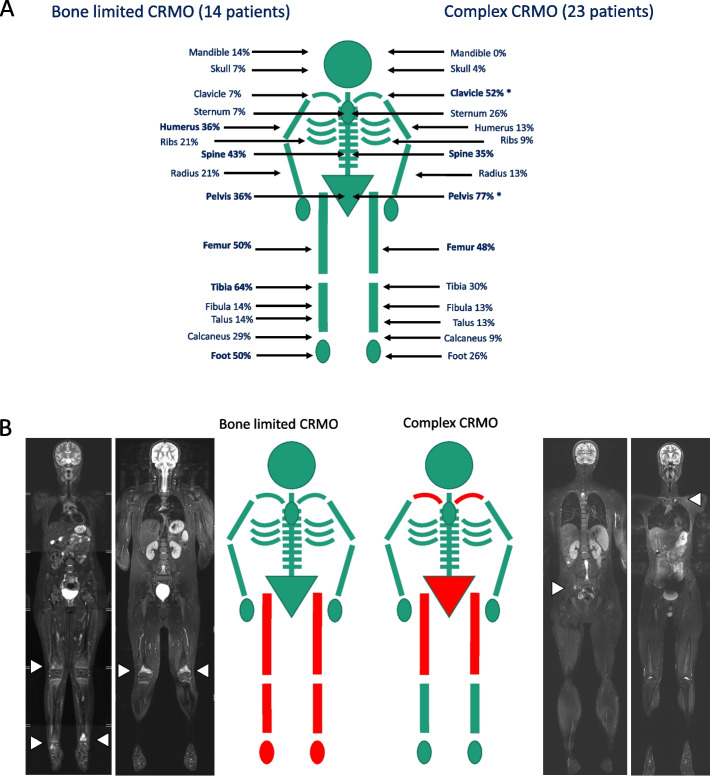
Distribution of CRMO lesions in bone limited CRMO patients (left) and complex CRMO patients (right). **A** Frequencies ≥35% are considered important (bold). *P*-values for each site are summarized in Table 4. Asterisk indicates significant difference. **B** Dominant patterns (red) of affected bones in bone limited CMRO (left) and complex CRMO (right) with illustrative cases of WB-MRI. Arrowheads indicate main affected sites

In the next part of our study, we aimed to compare bone limited and complex CRMO in children. A family history of autoimmune diseases was present in 71% of children with bone limited CRMO and in 65% of children with complex CRMO. No differences were found for blood markers like leukocyte and platelet counts, haemoglobin, and mean cell volume (MCV) (Fig. 4A). By contrast, inflammatory activity was significantly higher in complex than in bone limited disease (CRP, *p* = 0.018; ESR, *p* = 0.0064; Fig. 4B). Children with bone limited CRMO manifested disease slightly earlier (mean 105 ± 36 months) than did children with complex CRMO (mean 123 ± 38 months); however, the difference was statistically not significant (*p* = 0.1336). Nor did the number of lesions differ between the two groups (Fig. 4B). Interestingly, we found statistically significant differences in affected sites between the two groups. Whereas children with bone limited CRMO often displayed long-bone involvement, children with complex CRMO presented predominantly with involvement of clavicle and pelvis (clavicle, *p* = 0.011; pelvis, *p* = 0.038; Fig. 5A, B). Frequent and almost equal between both patient groups was femoral (50% in bone limited CRMO, 48% in complex CRMO) and spinal involvement (43% in bone limited CRMO, 35% in complex CRMO; Table 4).

**Table 4:** Distribution of lesions in patients with bone limited and complex CRMO

Affected site	Bone limited CRMO (no = 14)	Complex CRMO (***n*** = 23)	***P*** value
skull	1	1	1.000
mandible	2	0	0.137
scapula	1	1	1.000
clavicle	1	12	0.011
sternum	1	6	0.217
humerus	5	3	0.215
radius	3	3	0.653
ribs	3	2	0.346
spine	6	8	0.732
pelvis (incl. sacrum)	5	17	0.038
femur	7	11	1.000
tibia	9	7	0.086
fibula	2	3	1.000
talus	2	3	1.000
calcaneus	4	2	0.173
feet with digits	7	6	0.171

**Fig. 6:**
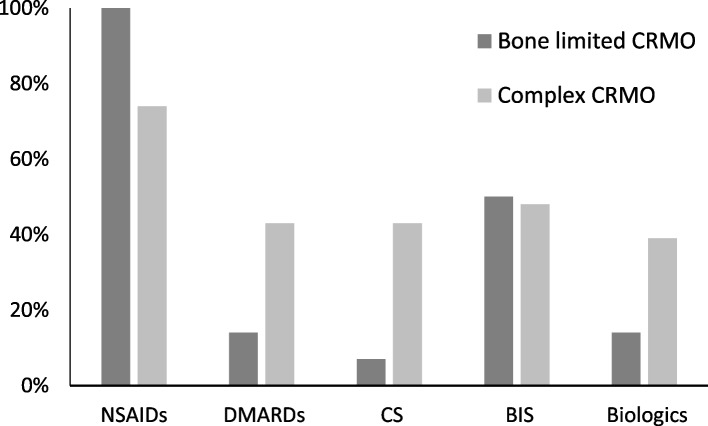
Treatments applied in patients with bone limited (dark grey) and complex (light grey) CRMO. NSAID - non-steroidal anti-inflammatory drugs, DMARDs – disease modifying antirheumatic drugs, CS – corticosteroids, BIS – bisphosphonates, TNFi – tumour necrosis factor α inhibitors

Treatment options used in our cohort are summarized in Fig. 6. All children with bone limited CRMO and 74% of those with complex CRMO received NSAIDs, including ibuprofen, naproxen, or diclofenac. Half of the patients were treated with bisphosphonates (pamidronate or zoledronate) because of spinal involvement or inefficacy of NSAIDs. The percentage of bisphosphonate treatment was similar for the bone limited and complex CRMO groups (respectively 43 and 50%), while spinal involvement was identified in respectively 43 and 35% of children. Children with complex CRMO more often received conventional synthetic DMARDs, corticosteroids, and biologic therapy (TNFi) (Fig. 6). Substantial improvement or disease remission was achieved in 27 patients (73%); 7 children (19%) still have active chronic or remittent disease. Three patients with complex CRMO were lost to follow-up due to non-compliance.

## Discussion

In this study, we focused on different phenotypes of CRMO. We observed that children with extraosseous involvement present with significantly increased inflammatory activity, have a distinct type of bone involvement, and more frequently require escalation to a second-line therapy. No differences in number of bone lesions and in age at disease onset were found.

Some earlier studies noticed phenotypic differences between CRMO patients and endeavoured to stratify their patients according to severity and disease course [7, 16, 23]. Wipff et al. defined three subgroups of CRMO patients [7]. Compared to the mild form, children with intermediate and severe phenotypes more frequently presented with extraskeletal co-morbidities, multifocal disease, and inflammatory syndrome. The more aggressive CRMO phenotype, with higher rates of multifocality, higher ESR, and predominant involvement of feet, tibia, pelvis, and humerus was described in children with extraosseous manifestation by Borzutzky and colleagues [6]. In accordance with these findings, O’Leary et al. demonstrated multifocality of lesions and low response rate to NSAIDs in their Irish cohort with a high prevalence of extraosseous involvement. We observed similar differences between children with and without extraosseous manifestations. Our findings further suggest that lesions in complex CRMO patients with affected extraskeletal tissues, particularly skin, gut, and joints, are differently distributed. Clavicular and pelvic lesions dominated in complex CRMO whereas lesions in metaphysis of long bones and foot were less frequent (Fig. 5B). The prevalence of pelvic involvement in patients with complex CRMO correlates with frequent affection of sacroiliac joints, one of the extraosseous features of complex CRMO. On the contrary, foot and long bones, specifically tibia, were prevalent sites of lesions in bone limited CRMO. In our group of patients, we noticed an unusually high incidence of IBD compared with other cohorts. In the subgroup of patients with IBD, the dominant osteolytic pattern comprises clavicle and pelvis. However, further studies on a larger cohort are required for generalisation of our observations and determination of predictive value of lesion distribution in CRMO subtypes.

Family history has an important, yet not fully understood role in CRMO. More than 50% of children in reported cohorts had family histories of autoimmune disorders, especially of those on the spondyloarthritis spectrum [6, 8]. In our cohort, extraosseous manifestations and autoimmune diseases in families exhibit similarities (Fig. 2). Arthritis, IBD, and psoriasis are conditions shared between the two groups. However, specific autoimmune diseases are not clustered within the families in any apparent pattern. For example, only half of the children with enteropathic CRMO in our cohort have a family history of IBD, and none of HLA-B27 positive patients had family history of HLA-B27 associated SpA or psoriasis. On the other hand, three relatives of patients with enteropathic CRMO presented with HLA-B27 positive SpA or psoriasis. Overall, the presence of HLA-B27 antigen in our cohort was twice as high as that in a general Central European population [24] and was not associated with the presence of sacroiliitis.

Arthritis or psoriasis is known to be associated with alteration of gut microbiota [25–27]. In a paediatric patient with enteropathic CRMO, successful deployment of a Crohn-disease exclusion diet was described in a recent report [28]. Thus, altered microbiome can represent an additional factor contributing to the development of enteropathic CRMO. However, in addition to those with the enteropathic form, we observed elevated FCP levels in 31% of our patients with active CRMO, indicating the presence of subclinical gut inflammation. Elevated FCP was found in four out of six HLA-B27 positive CRMO patients, but just one developed IBD during follow-up. Such observation was previously reported for patients with ankylosing spondylitis. IBD occurred more frequently in HLA-B27 negative patients [29].

Enteropathic form appears as a specific subtype of CRMO and in many aspects resembles SpA. In SpA, 5–10% of patients develop IBD, and further 70% present with subclinical gut inflammation [30, 31]. Recent genetic studies on SpA and IBD provided genetic support for this clinical observation and discovered several susceptibility genes shared among these two conditions. Indeed, a link between the SpA pathogenesis and defective gut mucosal immunity was suggested [32]. The retrospective character of this study prevented analysis of the microbiome in our patients. However, in our cohort, children with enteropathic CRMO frequently presented with severe phenotype and suffered from more than one extraosseous manifestation. Thus, a better understanding of the interplay between genetic predispositions, mirrored in family history of the probands, and environmental factors could promote our understanding of disease aetiopathogenesis and possibly shape future therapy strategies. For example, children with a higher risk of IBD may profit from methotrexate [33], an earlier switch to TNFi or from NSAIDs avoidance. Similarly, by identifying patients at high risk for development of psoriasis, including paradoxical disease during TNFi treatment, concomitant treatment with DMARDS can be encouraged [34, 35].

Even though our study has yielded several interesting findings, we must mention limitations that restrain us from offering general recommendations. First and foremost, the number of children with CRMO in this cohort is low. The retrospective approach in this study did not allow implementation of a uniform treatment protocol for all patients and a comprehensive evaluation of the treatment response between the two groups of CRMO. Importantly, our findings are in line with other studies indicating that CRMO is a heterogeneous disease [36, 37] and might be considered as an umbrella term for phenotypically similar disorders with potentially diverse etiopathogenetic backgrounds and sterile osteomyelitis as a dominant feature.

In conclusion, osteomyelitis as a dominant phenotypic feature of CRMO is accompanied by inflammation of other tissues in a substantial group of patients. The disease thus presents in two main forms, which we describe as ‘bone-limited’ and ‘complex’. An alternative concept of CRMO as a clinical continuum between these two distinctive forms urges the need of more structured and targeted therapeutic strategies, which reflect the heterogeneity of the disease. Thorough clinical stratification of patients in a multicentre study followed by genetic-epigenetic studies can promote early identification of high-risk patients, who could benefit from more aggressive treatment with early therapy escalation.

## Data Availability

The datasets used and/or analysed during the current study are available from the corresponding author on reasonable request.

## Abbreviations

CNO: Chronic non-bacterial osteomyelitis
CRMO: Chronic recurrent multifocal osteomyelitis
CRP: C-reactive protein
DIRA: Deficiency of interleukin-1 receptor antagonist
DMARD: Disease-modifying anti-rheumatic drug
FCP: Faecal calprotectin
HLA: Human leukocyte antigen
IBD: Inflammatory bowel disease
MRI: Magnetic resonance imaging
NSAID: Non-steroidal anti-inflammatory drug
SAPHO: Synovitis, acne, pustulosis, hyperostosis, osteitis
SpA: Spondyloarthritis
TNFi: Tumour necrosis factor inhibitor
WB-MRI: Whole-body MRI
